# Growth Axis *Somatostatin*, *Growth Hormone Receptor*, and *Insulin-like Growth Factor-1* Genes Express and Are Affected by the Injection of Exogenous Growth Hormone in *Chinemys reevesii*

**DOI:** 10.3390/genes14112032

**Published:** 2023-10-31

**Authors:** Rui-Lin Xie, Rui Liang, Yuan-Yuan Luo, Zhuo-Hao Ruan, Yi-Fu Li, Wen-Sheng Liu

**Affiliations:** 1 College of Marine Sciences, South China Agricultural University, Guangzhou 510642, China; m15876536141@163.com (R.-L.X.); 17334610172@163.com (Y.-Y.L.); 18376022717@163.com (Y.-F.L.); 2 Foshan Institute of Agricultural Science, Foshan 528251, China; rayliang2023@163.com; 3 Laboratory of Aquatic Sciences, Key Laboratory of Animal Nutrition and Feed Science in South China of Ministry of Agriculture and Rural Affairs, Guangdong Key Laboratory of Animal Breeding and Nutrition, Institute of Animal Science, Guangdong Academy of Agricultural Sciences, Guangzhou 510610, China; zhuohaoruan@163.com; 4 Guangdong Province Engineering Research Centre of Aquatic Immunization and Aquaculture Health Techniques, South China Agricultural University, Guangzhou 510642, China; 5 University Joint Laboratory of Guangdong Province, Hong Kong and Macao Region on Marine Bioresource Conservation and Exploitation, Guangzhou 510642, China

**Keywords:** *Chinemys reevesii*, growth hormone injection, *somatostatin*, *growth hormone receptor*, *insulin-like growth factor-I*

## Abstract

In this study, to explore the effect of growth hormone changes on the related genes and regulatory roles of the turtle, PCR amplification, real-time fluorescence quantitative analysis, and enzyme cutting technology were used to clone and sequence the *somatostatin* (*SS*) gene, *growth hormone receptor* (*GHR*), and *insulin-like growth factor-1* (*IGF-I*) sequence of *Chinemys reevesii*. The effects of human growth hormone on the mRNA expression of growth-axis-related genes *SS*, *GHR*, and *IGF-1* in different sexes were observed. The study of the *SS* gene in turtles using real-time fluorescence quantitative PCR showed that the *SS* gene was mainly expressed in the nervous system and the digestive system, with the highest expression found in the brain, while the *GHR* gene and the *IGF-I* gene were expressed in all tissues of *Chinemys reevesii*. The *SS* gene was expressed in the brain, pituitary, liver, stomach, and intestine, with the highest expression in the brain and the lowest expression in the liver. Within 4 weeks of the injection of exogenous growth hormone, the expression level of the *SS* gene in the brain of both sexes first increased and then decreased, showing a parabolic trend, and the expression level of the experimental group was lower than that of the control group. After the injection of growth hormone (GH), the expression of the *GHR* gene in the liver of both sexes showed a significant increase in the first week, decreasing to the control group level in the second week, and then gradually increasing. Finally, a significant level of difference in the expression of the *GHR* gene was reached at 3 and 4 weeks. In terms of the *IGF-I* gene, the changing trend of the expression level in the liver was the same as that of the *GHR* gene. After the injection of exogenous growth hormone, although the expression of the *SS* gene increased the inhibition of the secretion of the *GHR* gene by the Reeves’ turtle, exogenous growth hormone could replace the synthesis of GH and *GHR*, accelerating the growth of the turtle. The experiments showed that the injection of recombinant human growth hormone affects the expression of *SS*, *GHR*, and *IGF-1* genes, and promotes the growth of the Reeves’ turtle.

## 1. Introduction

Turtles are long-living animals with complicated body growth, and there is both determinate and indeterminate body growth in this group [1,2,3,4]. The main difference is in the ability to continue growth throughout life in indeterminate growers, while the determinate ones cease their skeletal growth typically close to sexual maturation [5,6,7]. There are many kinds of turtles, roughly divided into three kinds: tortoise, terrapin, and semi-water turtle. *Chinemys reevesii* is a freshwater turtle belonging to Chordata, Reptilia, Testudoformes, and Geoemydidae that is mainly distributed in subtropical and temperate regions, such as China, Japan, and South Korea. Generally, turtles are long-living and slow-growing animals, making them suitable for use as animal models to study growth [8,9]. However, determining why turtles grow slowly remains an unsolved problem. Moreover, the causal relationship between growth retardation and gene expression patterns related to the growth axis is still unclear. Growth hormone and *somatostatin* (*SS*) are key factors that regulate the growth and development of an animal [10]. To date, the *SS* gene expression pattern of the Reeves’ turtle remains unknown. In previous studies on the hypophysis microstructure, adenohypophysis ultrastructure, and blood biochemical indexes [11,12], it was found that fewer granules are secreted by the GH cells of the hypophysis of turtles compared with other animals. 

*SS* is a low molecular basic peptide secreted in the hypothalamus, and was first discovered by British scientists in 1968 [13]. After a series of experiments, such as separation, purification, and identification, Brazeau et al. extracted the substance successfully, determined its structure as a cyclic 14-peptide (*SS*-14), and officially named it as *SS* [14]. Pradayrol et al. later found that *SS*-28 and *SS*-25 of the *SS*-14 amino-terminal extension were both encoded by the same gene in mammals [15,16]. Since the discovery of *SS*, it has been detected in various vertebrates, mainly in the nervous system and the digestive system. It has many functions, such as inhibiting the activity of the nervous system and the spontaneous electrical activity of the brain [17]. An injection of *SS* into the animal brain and spinal cord can effectively relieve pain [18]. In the clinic, *SS* can inhibit the production of antibodies via B lymphocytes and can also inhibit the release of inflammatory factors [19,20,21,22] to resist the inflammatory reaction. In addition, it can inhibit the exocrine function of the gastrointestinal tract and pancreas, the release of gastrointestinal hormones and proteases, gastrointestinal peristalsis, and the repair of gastrointestinal mucosa [23]. In terms of regulating animal growth, growth hormone and *insulin-like growth factor-1* (*IGF-I*) can promote animal growth, while *SS* can inhibit the release of various hormones, such as growth hormone (GH), thyroid-stimulating hormone (TSH), gonadotropin-releasing hormone (GnRH), etc. [24,25], which play a negative feedback role in the growth axis, and work with growth hormone and *IGF-I* to regulate the growth and metabolism of animals. Based on this function, *SS* can effectively inhibit the growth of tumors [26,27,28]. Mclean and Donaldson found that an exogenous injection of growth hormone extracted from mammals can promote the growth of amphibians and reptiles [29]. Therefore, in this study, we injected turtles with human growth hormone to observe the expression of *SS* mRNA, *GHR* mRNA, and *IGF-I* mRNA related to the growth axis of the Reeves’ turtle of different sexes, to explore the effect of growth hormone on related genes and its regulatory role, and to determine the main reason for the slow growth of turtles. 

## 2. Materials and Methods

### 2.1. Experimental Animal

In this experiment, male and female turtles were identified and 100 healthy first-year young turtles were selected. There are two main criteria used for the discrimination of sex in *C. reevesii*. One is the turtle’s belly nail—the female’s is flat and the pattern is denser, while the male’s is concave and the pattern is sparser. The other is the turtle’s tail—the female tail is slender and close to the anus, while the male tail is shorter and far from the anus. The experimental animals were from Guangdong Zhangcheng Ecological Agriculture Investment Co., Ltd (Guangdong, China). with a weight of 80–100 g. The water temperature was kept at 24–26 °C. The change in the water temperature in the aquaculture pond was affected by the day/night cycle, but due to the thermal characteristics of the water itself, the change in water temperature in the aquaculture pond was much smaller than the air temperature. In addition, the water level was 1 cm higher than the back of the young turtle. The amount of feed was approximately 3% of the total body weight of the young turtle. The *C. reevesii* feed used in the experiment was purchased from Shanghai Derui Feed Co., Ltd (Shanghai, China). The main ingredients of the feed were crude protein 30%, total phosphorus 0.8%, lysine 1.1%, crude fiber 6.5%, 13% crude ash, 3% calcium, 3% crude fat, and 10% water. The Animal Care Committee of South China Agriculture University (Guangzhou, China) approved the current study under the trial registration number G027, and the research was accomplished according to the Experimental Animal Management Law of China.

### 2.2. Sample Preparation

The growth hormone used in the experiment was sourced from Shanghai Shushi Biotechnology Co., Ltd (Shanghai, China). The male and female turtles in the experimental group were injected with growth hormone from 10 μg/g via by intraperitoneal injection once a week, lasting 4 weeks. The Reeves’ turtles in the control group were injected with same volume of PBS, and the other breeding conditions were consistent with the experimental group. Every week, male and female turtles were randomly selected for dissection once a week, and seven organs (brain, hypophysis, liver, spleen, kidney, stomach, and intestine) were placed in liquid nitrogen, and then transferred to the refrigerator at −80 °C for preservation.

### 2.3. Tissue Collection and Isolation of RNA

In order to determine the expression pattern of the tissue-specific gene, seven organs and tissues were removed from the turtles: liver, spleen, kidney, brain, hypophysis gland, stomach, and intestine. The total RNA was extracted with the Eastep Super RNA Extraction Kit (Promega, Beijing, China), and the cDNA was then synthesized via reverse transcription from 1 μg RNA (OD260/OD280) with the GoScript^TM^ RT Mix Kit (Promega, Beijing, China).

### 2.4. Cloning and DNA Sequencing of the SS Gene of the Reeves’ Turtle

The NCBI website was used to query the cDNA sequence of the *SS* gene of the species close to the Reeves’ turtle in GenBank, and DNAMAN software (LynnonBiosoft, American) was used to determine its conservative region. Primer Premier 6.0 software was employed to design specific nucleotide sequence primers for the *SS* gene of the turtles. Accordingly, the forward and reverse primers were 5′-GGCAAACAGGAACTGGC-3′ and 5′-ACAGTCTTCGGCTCGTGTCGTG-3′, respectively. PCR was performed by using the DNA polymerase of 2 × TransStart^®^ KD Plus PCR SuperMix (TransGen Biotech, Beijing, China) under the following conditions: an initial denaturing cycle at 94 °C for 5 min, followed by 30 cycles of amplification consisting of denaturation at 94 °C for 30 s, annealing at 52 °C for 1 min, and extension at 68 °C for 1 min. A final extension at 68 °C for 10 min was added after the last cycle. The PCR product was run using 1% (*w*/*v*) agarose gel electrophoresis with 30 mM Tris-acetate buffer (pH 8.0) containing 1 mM EDTA and visualized using ethidium bromide (0.5 µg/mL). The PCR product was linked with a pMD18-T simple vector (Takara Bio, Beijing, China) to form a recombinant plasmid. The recombinant plasmid DNA was introduced into DH5α-sensitive *Escherichia coli* for culture and amplification. Sequencing was then carried out by Qingke Biotechnology Co., Ltd. (Beijing, China). The cloned *SS* PCR product was expected to be 294 base pairs (bp).

### 2.5. RT-PCR

The nucleotide sequence of the *SS* gene of turtles was obtained via sequencing, and RT-PCR primers were designed. The forward and reverse primers were 5′-GGCAAACAGGAACTGGC-3′ and 5′-TTAGCCGATCGCTCCAACTC-3′, respectively. The gene expression of each turtle’s organ was detected using the newly acquired *SS* sequence, GHR gene, and *IGF-I* gene obtained from previous experiments in the laboratory. This was carried out under the following conditions: an initial denaturing cycle at 95 °C for 2 min, followed by 40 cycles of amplification at 95 °C for 15 s, the annealing temperature was then adjusted according to primer temperature for 1 min, the extension was at 72 °C for 1 min, the fluorescence signal was collected at 72 °C, and dissolution curve analysis was added to the reaction conditions at the end of the cycle. In this study, β-actin was used as the internal reference gene of RT-PCR. 

### 2.6. Statistical Analysis

Considering the influence of the size, age, and kinship of the experimental animals, first-year turtles of the same size were selected for the experiment. The turtles selected in this experiment were full-sibs. The data in this study were expressed as the mean ± SEM (standard error of mean; the standard error of the sample mean was used to measure the gap between the sample mean and the population mean). One-way ANOVA was used to test the differences between the groups. The difference was significant (*p* < 0.05). All the statistics were calculated using Graphpad Prism 6 (Graphpad Software Inc., San Diego, CA, USA), and the data were logarithmically transformed. 

## 3. Results

### 3.1. Tissue Distribution of SS Gene Expression in the Reeves’ Turtle

**Figure 1:**
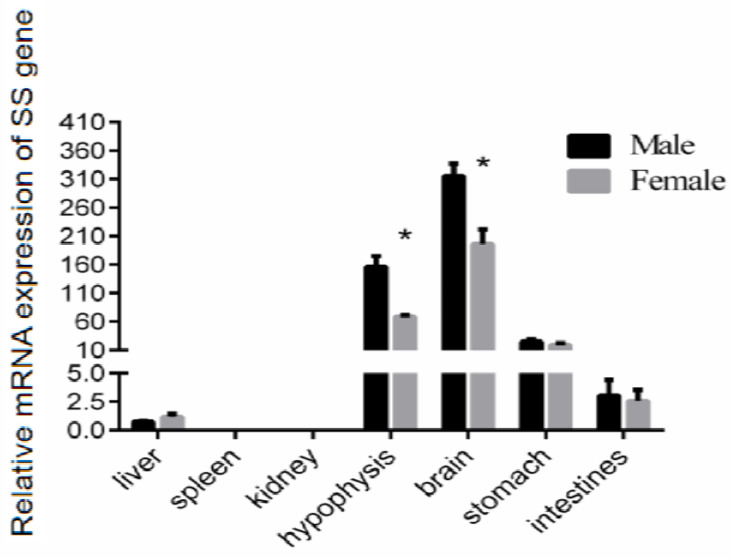
Relative gene expression of *SS* in male and female Reeves’ turtles. Total RNA from different tissues of three Reeves’ turtles was used to perform the real-time PCR. Each sample was performed in duplicate. The expression levels of the *SS* gene in the Reeves’ turtles were normalized to the expression of the β-actin gene. Data were processed using Graphpad Prism 6 (Graphpad Software Inc., San Diego, CA, USA), and were subjected to variance analysis (ANOVA) using the Open GL Mathematics (GLM) process. Different superscripts signify significant differences (* *p* < 0.05). Data are expressed as mean ± standard error of the mean (n = 3).

To determine the expression of the *SS* gene in the liver, spleen, kidney, brain, hypophysis, stomach, and intestinal tissue of the Reeves’ turtle, we cloned part of the *SS* gene for subsequent research. The qRT-PCR results showed that the expression level of *SS* mRNA in the brain was the highest, whether in male or female Reeves’ turtles. The expression level of *SS* in the hypophysis was nearly half that of the brain. The expression of *SS* in these two tissues was higher than in other tissues. Compared with the *SS* mRNA expression in the brain, the *SS* gene expression levels in the stomach, intestine, and liver tissues were approximately 7.9%, 1%, and 0.2%, respectively. Interestingly, the expression levels of the *SS* gene in the male brain and the hypophysis were significantly higher than those in the female (*p* < 0.05). However, the expression of *SS* in other tissues shared no sex difference. The transcription of *SS* could not be detected either in the male/female spleen or kidney (Figure 1). 

### 3.2. Modulation of SS Gene Expression in the Brain under Periodic Injection of Growth Hormone

**Figure 2:**
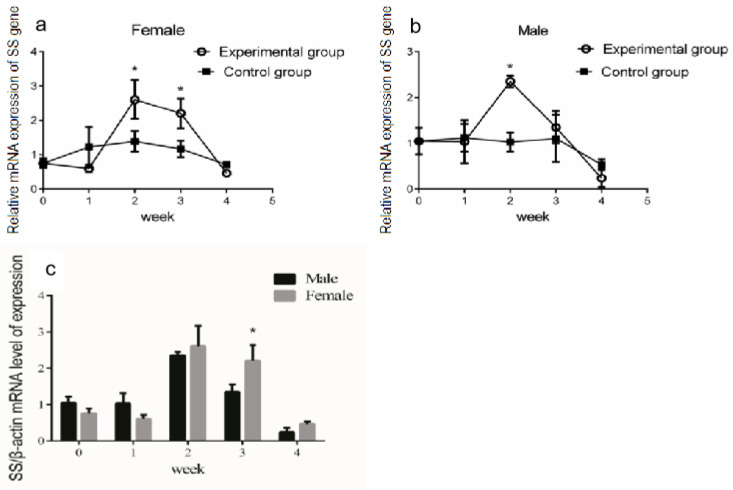
Modulation of *SS* expression in the brain after injection of GH. The turtles were divided into two groups, the experimental group and the control group, which contained the same number of male and female turtles. We first measured the body weight of the turtles. Male or female Reeves’ turtles in the experimental group were injected with a certain amount of growth hormone weekly. The turtles to be injected were weighed and were injected according to the standard 10 μg/g. Meanwhile, male or female Reeves’ turtles in the control group were injected with same volume of PBS. (**a**) Relative *SS* gene mRNA expression in the brain tissue of male Reeves’ turtle. (**b**) Relative *SS* gene mRNA expression in the brain tissue of female Reeves’ turtle. (**c**) Sex differences regarding *SS* gene expression in the brain after four weeks of GH injection. β-actin was used as the internal gene of qRT-PCR. Data were processed using Graphpad Prism 6 (Graphpad Software Inc., San Diego, CA, USA), and were subjected to variance analysis (ANOVA) using the GLM process. Different superscripts signify significant differences (* *p* < 0.05). Data are expressed as mean ± standard error of the mean (n = 3).

The growth hormone/insulin-like growth factor (GH/IGF) axis is an essential endocrine system regulating vertebrate growth [30]. However, the effect of growth hormone on the expression of *SS* in Reeves’ turtles was still unclear. Given that the highest mRNA expression level of *SS* was found in the brain, we analyzed the modulation of *SS* expression in the brain after periodic injections of growth hormone. The qRT-PCR results showed that the *SS* mRNA expression in the brain tissue did not change significantly in the first week after injection (*p* > 0.05, effect size of Cohen d = 0.2). After two weeks of continuous injection, the expression of *SS* in the brain tissue of female turtles increased significantly (*p* < 0.01, effect size of Cohen d = 0.8) (Figure 2a). After two or three weeks of continuous injection, the expression of *SS* mRNA in the male turtle brain increased significantly (*p* < 0.01, effect size of Cohen d = 0.8) (Figure 2b). Interestingly, in the fourth week of continuous injection, the *SS* gene expression in the brain tissue of male and female turtles shared no significant difference compared with the control group (*p* > 0.05, effect size of Cohen d = 0.2). Collectively, the *SS* gene expression in the brain of male and female turtles presented a trend of increasing first, and then decreasing. Further data analysis revealed that, in the third week of continuous GH injection, expression of the *SS* gene in the brain tissue of female turtles was significantly higher than that of males (*p* < 0.05) (Figure 2c). 

### 3.3. Effect of Exogenous Growth Hormone on SS Gene Expression in Tissues of the Reeves’ Turtle

**Figure 3:**
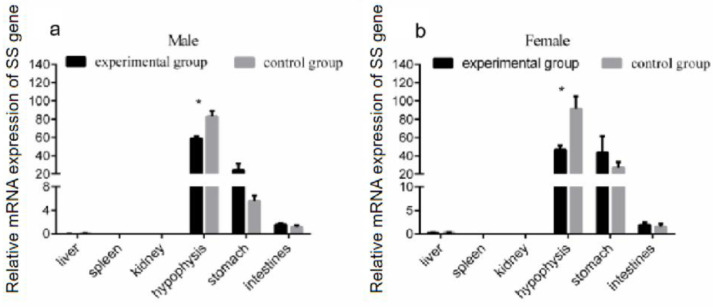
Expression of the *SS* gene in each organ after 4 weeks of growth hormone injection. The turtles were divided into two groups, the experimental group and the control group, containing the same number of male and female turtles. We first measured the body weight of the turtles. Male or female Reeves’ turtles in the experimental group were injected with a certain amount of growth hormone weekly. The turtles to be injected were weighed and were injected according to the standard 10 μg/g. Meanwhile, male or female Reeves’ turtles in the control group were injected with same volume of PBS. (**a**) The mRNA expression of the *SS* gene in the male Reeves’ turtle after four weeks of GH stimulation. (**b**) The mRNA expression of the *SS* gene in the female Reeves’ turtle after four weeks of GH stimulation. β-actin was used as the internal gene of qRT-PCR. Data were processed using Graphpad Prism 6 (Graphpad Software Inc., San Diego, CA, USA), and were subjected to variance analysis (ANOVA) using the GLM process. Different superscripts signify significant differences (* *p* < 0.05). Data are expressed as mean ± SEM (n = 3).

Given the modulation of *SS* gene expression in the brain through GH injection, we further analyzed whether a similar effect existed in other organs of the Reeves’ turtle. The turtles were divided into two groups: the experimental group and the control group. The male and female Reeves’ turtles in the experimental group were injected weekly with a certain amount of growth hormone on Mondays for four weeks. The Reeves’ turtles in the control group were injected with the same volume of PBS. Four weeks after the injection of GH, the mRNA expression of the *SS* gene in different tissues of male and female Reeves’ turtles was analyzed via qRT-PCR. Compared with the control group, the expression of the *SS* gene mRNA in the hypophysis decreased significantly (*p* < 0.05, effect size of Cohen d = 0.5) (Figure 3a,b). The expression of the *SS* gene in the stomach and intestinal tissues did not change significantly under the stimulation of GH (*p* > 0.05, effect size of Cohen d = 0.2). In both male and female turtles, the expression of *SS* in the liver, spleen, and kidney were so low that they were almost undetectable. Interestingly, when facing the GH stimulation, the mRNA expression profile of *SS* had a similar response trend in different tissues both in male and female turtles.

### 3.4. Modulation of GHR Gene Expression in the Liver under Periodic Injection of Growth Hormone

**Figure 4:**
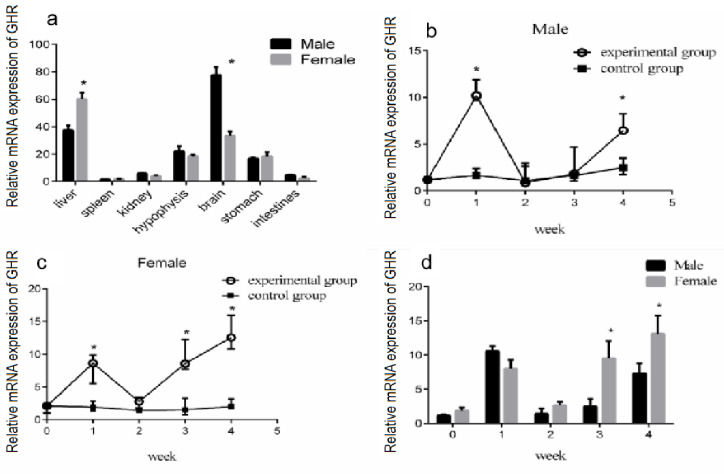
Effect of GH stimulation on *GHR* gene expression in the liver. The mRNA expression of *GHR* in different organs of the Reeves’ turtle and the effect of GH stimulation on *GHR* expression in the liver. (**a**) The mRNA expression of *GHR* in different organs of the Reeves’ turtle. The turtles were divided into two groups, the experimental group and the control group, containing the same number of male and female turtles. We first measured the body weight of the turtles. Male or female Reeves’ turtles in the experimental group were injected with a certain amount of growth hormone weekly for four weeks. The turtles to be injected were weighed and injected according to the standard 10 μg/g. Meanwhile, male or female Reeves’ turtles in the control group were injected with same volume of PBS. (**b**) Relative *GHR* gene mRNA expression of male Reeves’ turtle liver under GH stimulation. (**c**) Relative *GHR* gene mRNA expression of female Reeves’ turtle liver under GH stimulation. (**d**) Sex differences in *GHR* gene expression in the liver after four weeks of GH injection. β-actin was used as the internal gene of qRT-PCR. Data were processed using Graphpad Prism 6 (Graphpad Software Inc., San Diego, CA, USA), and were subjected to variance analysis (ANOVA) using the GLM process. Different superscripts signify significant differences (* *p* < 0.05). Data are expressed as mean ± SEM (n = 3).

*GHR* is an essential endocrine regulator of the growth axis of GH/*IGF-I*. The biological role of growth hormone is mediated by *GHR* [8]. In the liver of fish and other vertebrates, the expression level of *GHR* was found to be high [8,31,32]. We measured the mRNA expression of the *GHR* gene in the liver, spleen, kidney, brain, hypophysis, stomach, and intestine of turtles via the qRT-PCR method. The *GHR* gene was highly expressed in the liver, brain, stomach, and hypophysis of the Reeves’ turtle, but the abundance of *GHR* mRNA in the spleen, kidney, and intestines was low (Figure 4a). 

To further clarify the effect of GH stimulation on *GHR* gene expression in turtle liver, the turtles were divided into two groups, the experimental group and the control group, containing the same number of male and female turtles. We first measured the body weight of the turtles. Male or female Reeves’ turtles in the experimental group were injected with a certain amount of growth hormone weekly for four weeks. The turtles to be injected were weighed and were injected according to the standard 10 μg/g. Meanwhile, male or female Reeves’ turtles in the control group were injected with the same volume of PBS. In the first week of the GH injection experiment, the mRNA expression of *GHR* was significantly increased in both the male and female Reeves’ turtles in the experimental group (*p* < 0.05). However, during the second and third week of the GH injection experiment, the *GHR* expression in the male turtle liver in the experimental group was decreased, and there was no significant difference between the control group and the experimental group (*p* > 0.05, effect size of Cohen d = 0.2) (Figure 4b). Meanwhile, in the second week of the GH injection experiment, the *GHR* expression in the female turtle liver in the experimental group was decreased, and there was no significant difference with the control group (*p* > 0.05, effect size of Cohen d = 0.2) (Figure 4c). Interestingly, in the fourth week of the GH injection experiment, the *GHR* gene expression in the male turtle liver was increased significantly again (*p* < 0.01, effect size of Cohen d = 0.8) (Figure 4b). In the third and fourth weeks of GH injection, the *GHR* gene expression of the female turtle liver was significantly higher than that of the control group (*p* < 0.01, effect size of Cohen d = 0.8) (Figure 4c). The *GHR* gene expression in the liver of male and female turtles presented a change trend of increasing first, and then decreasing, but eventually increasing again. Further data analysis revealed that in the third and fourth week of GH injection, the expression of the *GHR* gene in the female turtle liver was significantly higher than that of the male turtle (*p* < 0.05) (Figure 4d).

### 3.5. Modulation of GHR mRNA Expression in Different Tissues of Reeves’ Turtles after Four Weeks of GH Injection

**Figure 5:**
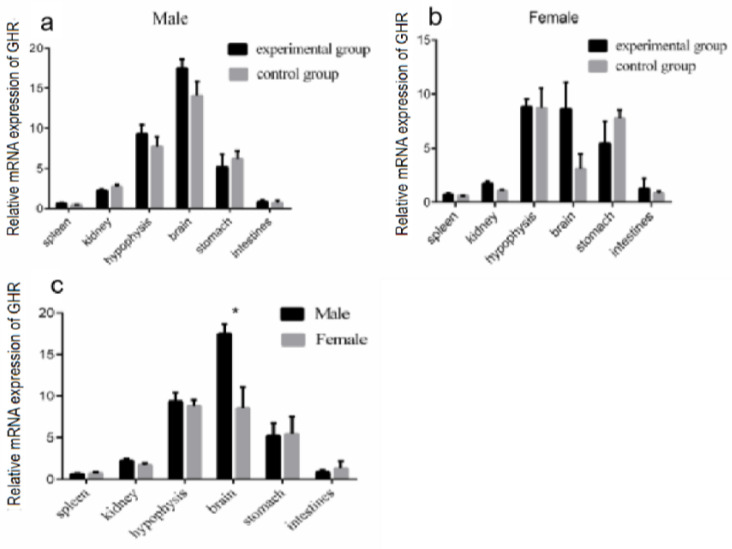
Effect of four weeks of GH stimulation on *GHR* expression. Expression of *GHR* in different tissues of the Reeves’ turtle after four weeks of GH injection. (**a**) The mRNA expression of the *GHR* gene in the male Reeves’ turtle after four weeks of GH stimulation. (**b**) The mRNA expression of the *GHR* gene in the female Reeves’ turtle after four weeks of GH stimulation. (**c**) The mRNA expression of the *GHR* gene in each tissue of female and male Reeves’ turtles. β-actin was used as the internal gene of qRT-PCR. Data were processed using Graphpad Prism 6 (Graphpad Software Inc., San Diego, CA, USA), and were subjected to variance analysis (ANOVA) using the GLM process. Different superscripts signify significant differences (* *p* < 0.05). Data are expressed as mean ± SEM (n = 3).

In order to clarify the effect of GH injection on *GHR* mRNA expression in different organs, the modulation of *GHR* expression in the Reeves’ turtle was detected after four weeks of GH injection. The *GHR* mRNA expression in different organs of male and female Reeves’ turtles did not change significantly after four weeks of GH stimulation (*p* > 0.05) (Figure 5a,b). The data analyzed with regard to sex differences showed that the *GHR* mRNA expression in the male turtle brain was significantly higher than that in the female turtle brain (*p* < 0.05) (Figure 5c).

### 3.6. Effect of Periodic Exogenous GH Injection on IGF-I Gene Expression in the Liver

**Figure 6:**
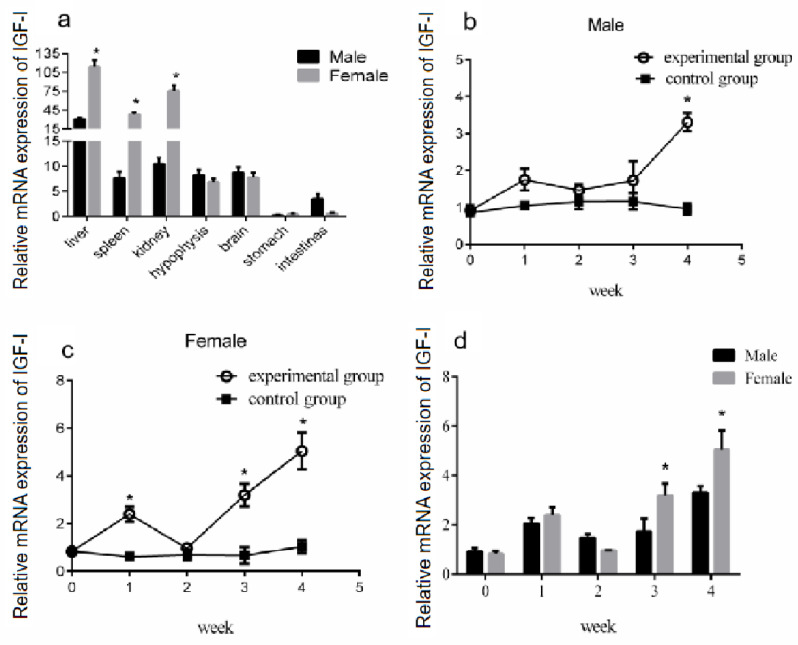
Effect of GH stimulation on *IGF-I* gene expression in the liver. (**a**) The mRNA expression of the *IGF-1* gene in each tissue of female and male Reeves’ turtles. (**b**) The mRNA expression of the *IGF-1* gene in the liver of the male Reeves’ turtle after four weeks of GH stimulation. (**c**) The mRNA expression of the *IGF-1* gene in the liver of the female Reeves’ turtle after four weeks of GH stimulation. (**d**) The mRNA expression of the *IGF-1* gene in the liver of the female and male Reeves’ turtles. The expression of the *IGF-I* gene in different tissues of the Reeves’ turtle. The average expression in the male liver is the highest at 30.72, while the expression in the stomach is the lowest at 0.28. The average expression in the female liver is the highest at 113.60, while the expression in the stomach is the lowest at 0.47. Different superscripts signify significant differences (* *p* < 0.05). Data were processed using Graphpad Prism 6 (Graphpad Software Inc., San Diego, CA, USA), and expressed as the mean ± SEM. Variance analysis was operated using GLM procedures, based on at least three replicates for each treatment.

*IGF-I* is a hormone that is fundamental to reproduction and lifespan, as well as to cell growth, differentiation, migration, and survival [33]. In vertebrates, it is produced both centrally by the liver and peripherally by diverse tissues [34]. To verify the effect of GH stimulation on *IGF-I* gene expression in the liver of male and female Reeves’ turtles, we first measured the expression of the *IGF-I* gene in the liver, spleen, kidney, brain, hypophysis, stomach, and intestine. The qRT-PCR data showed that *IGF-I* mRNA was expressed in different organs of the Reeves’ turtle. There are differences in the expression of *IGF-I* mRNA between the male and female. The *IGF-I* mRNA expression levels in the liver, kidney, and spleen of the female turtle were significantly higher than those of the male turtle (*p* < 0.05) (Figure 6a). However, the highest mRNA expression of *IGF-I* was found in both the male and female liver.

### 3.7. Changes in IGF-I Gene mRNA Expression in the Liver after Periodic Injection of Growth Hormone

**Figure 7:**
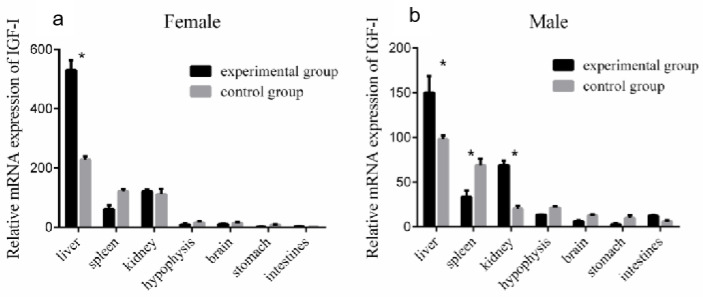
Expression of the *IGF-I* gene mRNA in each organ after 4 weeks of injection of growth hormone. (**a**) Compared with the control group, the expression of the *IGF-I* gene mRNA in the liver and kidney of the male experimental group increased significantly, while the expression in the spleen decreased significantly. (**b**) Compared with the control group, the expression of the *IGF-I* gene mRNA in the female experimental group increased significantly. Data were processed using Graphpad Prism 6 (Graphpad Software Inc., San Diego, CA, USA), and expressed as the mean ± SEM. Variance analysis was operated using GLM procedures, based on at least three replicates for each treatment. * *p* < 0.05 compared between the two indicated constructions.

To study the effect of GH injection on the expression of liver *IGF-I*, Reeves’ turtles in the experimental group were injected with a certain amount of growth hormone weekly for four weeks. Meanwhile, the turtles in the control group were injected with the same volume of PBS. After the injection of growth hormone, the expression of *IGF-I* mRNA in the liver of males and females in the control group remained basically stable, and the changing trend of *IGF-I* mRNA expression levels in the male and female liver were similar: the expression increased in the first week of the experiment, decreased in the second week, and continuously increased in the third and fourth weeks of the experiment. The expression of mRNA in the experiment group of the male turtle was significantly higher than that in the control group at the fourth week (*p* < 0.01, effect size of Cohen d = 0.8), while the expression in the female group was significantly higher than that in the control group at the first, second, and fourth weeks (*p* < 0.05, effect size of Cohen d = 0.5). The expression of *IGF-I* mRNA in the liver of male turtles in the experimental group was only slightly higher than that of female turtles in the second week, while that of female turtles was higher than that of male turtles in the first, third, and fourth weeks, with the difference significant in the third and fourth weeks (*p* < 0.05). The results are shown in Figure 6. Four weeks after the injection of growth hormone, the expression of the *IGF-I* gene mRNA in the liver and kidney of the male turtles in the experimental group was significantly higher than that in the control group (*p* < 0.05, effect size of Cohen d = 0.8), while the expression in the spleen was significantly lower than that in the control group (*p* < 0.05, effect size of Cohen d = 0.8), and the expression levels in the hypophysis, brain, and stomach were slightly lower than those in the control group (*p* > 0.05, effect size of Cohen d = 0.2). The expression of the *IGF-I* gene mRNA in the liver of the female turtles was significantly higher than that in the control group (*p* < 0.05, effect size of Cohen d = 0.8), and expression in the spleen was slightly lower than that in the control group (*p* > 0.05, effect size of Cohen d = 0.2). The results are shown in Figure 7.

## 4. Discussion

Under natural conditions, the growth rate of the Reeves’ turtle is relatively slow, being slow at 1–2 years, accelerating at 3–4 years, and reaching sexual maturity at 5–6 years. Female turtles grow faster than male turtles. In the 6th year of sexual maturity, female turtles weigh about 400 g, while male turtles weigh approximately 250 g [35]. Although the growth speed of turtles is slower than that of other vertebrates, their growth is also controlled by the growth hormone (GH)—*insulin-like growth factor-1* (*IGF-I*) system. *SS* mainly inhibits the growth of animals by reducing the release of hypophysis growth hormone [10]. As a type of inhibiting hormone required for regulating the stable growth of animals, *SS* can prevent animals from being damaged when they grow too fast. For example, because of the over-secretion of GH, the growth and development of patients with gigantism will be reduced, causing side effects such as low resistance and a short lifespan [36]. Therefore, *SS* plays an indispensable role in the growth axis of animals. In this study, β-actin was used as the internal reference gene of RT-PCR. The expression levels of the *SS* gene mRNA in the brain, hypophysis, liver, spleen, kidney, stomach, and intestine were analyzed. *SS* gene mRNA was highly expressed in the nerve and digestive tissues of the Reeves’ turtle, with the highest expression found in the brain. This is consistent with the research results regarding *SS* gene expression in humans, rats, fish [37,38,39], and other animals, but the expression amount in other tissues was not found to be the same. In this experiment, no expression of *SS* mRNA was found in the spleen or kidney of the Reeves’ turtle; however, the expression of *SS* mRNA has been detected in the spleen of fish [38], and also in the kidney of Chinese sturgeon [39]. According to a comprehensive analysis of the above results, the expression of *SS* gene mRNA is different among different species, but the expression of *SS* gene mRNA in the brain of different organisms is the highest, indicating that *SS* gene mRNA is expressed in different animals mainly in the brain, which is the main regulatory organ governing the release of the *SS* gene to control the growth and development of animals. However, due to the different expression characteristics, growth rules, and lifestyles of various animals, there may be differences in the expression of *SS* gene mRNA among the different organs of different animals. In this study, the relative expression of *SS* gene mRNA in the nervous system of male turtles was higher than that of female turtles. Combined with the above results, it is suggested that the growth speed of male and female turtles in the natural state is inconsistent, which may be caused by the difference in *SS* gene expression between male and female individuals. It was found that the expression abundance of growth hormone can affect the expression of *SS*. After Kamegai et al. excised the hypophysis gland of mice so that they could not secrete growth hormone by themselves, the concentration of *SS* decreased. When exogenous growth hormone was then injected, the concentration of *SS* in the mice began to increase again [40]. In this experiment, through a continuous injection of growth hormone, a large amount of the *SS* gene was expressed in both female and male turtles to balance their growth speed. Male turtles had strong adaptability to the growth hormone. After two weeks of injection, the turtles adapted to the injection amount of exogenous growth hormone, and the expression of *SS* gene mRNA in the brain began to decrease, which was similar to the natural state. However, the adaptability of female turtles to exogenous growth hormone was weak, and the expression of *SS* gene mRNA in the brain was higher and lasted longer than that in males, which indicates that the female is more sensitive to growth hormone. Therefore, it is speculated that the reason female turtles grow faster than male turtles under natural conditions may be due to the sensitivity of the female growth hormone.

Through experiments, we found that the *GHR* gene can be expressed in all tissues of the Reeves’ turtle, which is consistent with the expression in chickens, fishes, rats [41,42,43], etc. The expression of the *GHR* gene seriously influences the effect of growth hormone. When the expression of the *GHR* gene is high, it can combine with growth hormone to promote the growth of the organism. However, when the expression of the *GHR* gene is inadequate, growth hormone cannot combine with *GHR*, resulting in the organism producing negative feedback regulation, and inhibiting the growth and development of the organism. The *GHR* protein encoded by chickens with dwarfism is abnormal because of the mutation of the *GHR* gene, which cannot combine with growth hormone, resulting in growth retardation [44]. It was found that the expression of the *GHR* gene was different in different organs of animals at different periods. The expression of the *GHR* gene in the kidney of rabbits was the highest in the fetal period, while the expression of the *GHR* gene in the heart, liver, muscle, and other tissues was low. However, the expression of the *GHR* gene in the kidney was unchanged in the second to sixth month, but it was largely expressed in the heart, liver, muscle, and other tissues [45]. According to the above results, the developmental changes in *GHR* gene mRNA in different species and tissues will vary, but the highest expression level in the liver suggests that there may be differences in the expression regulation mechanism in different tissues. Combined with the results of this study, *GHR* gene mRNA expression in the brain of the male Reeves’ turtle was found to be the highest, followed by that in the liver. In the female, the expression of *GHR* gene mRNA was the highest in the liver, followed by that in the brain. This indicates that the male and female development of the Reeves’ turtle is not synchronous; the male development is slower than that of the female. The highest level of mRNA expression indicates that all the organs needed for survival are mature and begin to concentrate on the growth stage. Meanwhile, the highest level of *GHR* gene mRNA expression occurring in the male brain indicates that the brain development of the male is not perfect at this stage, so it is necessary to concentrate on the development of the brain to control the release of various hormones. This difference under natural conditions may be one of the reasons for the asynchronous growth of the male and female turtle. In this study, *GHR* gene mRNA expression in the liver of male and female turtles changed after the injection of human growth hormone. In the first week, the mRNA expression of the *GHR* gene in the livers of males and females increased rapidly, and was significantly higher than that in the control group. In the second week, the mRNA expression of the *GHR* gene in the livers of both turtles decreased to a level similar to that in the control group, and continued to increase at the third and fourth weeks, suggesting that the sensitivity of the livers of the Reeves’ turtle to exogenous growth hormone was higher at the first injection of growth hormone. The expression of mRNA increased rapidly. When injected twice, the body had adapted to the exogenous substance, so the expression of *GHR* gene mRNA in the liver was self-regulated to be consistent with that found under natural conditions. However, with the continuous injection of growth hormone, the concentration of growth hormone in the body increased, so that the mRNA expression of the *GHR* gene in the liver was increased, and began to promote the growth of the Reeves’ turtle.

*IGF-I* is one of the important hormones regulating the growth and development of animals. It is generally believed that *IGF-I* plays a leading role in the growth of animals. *IGF-I* can be synthesized by many tissues and organs in animals, while the main source of *IGF-I* in circulation is the liver [46]. In this experiment, we found that the *IGF-I* gene is widely distributed in various tissues of the Reeves’ turtle, and the *IGF-I* gene content in the liver is the highest. This result is consistent with that found in pigs, sheep, and *Oreochromis mossambicus* [47,48,49]. Although the expression level of the *IGF-I* gene is the same among all types of animals, the expression level of *IGF-I* in different tissues of different sexes of the same animal also varies. Combined with the results of this study, it was found that the expression level of *IGF-I* gene mRNA in the liver of the male and female Reeves’ turtle was the highest among all the tissues, but the expression level of the *IGF-I* gene was different between them. The expression of mRNA in the female turtle liver, spleen, and kidney was higher than that in the male, suggesting that the growth rate of females in the first year may be faster than that in males. Further, the tissues related to detoxification and metabolism, such as the spleen and kidney, develop better, and females in the first year can adapt to harsh environments better than the males. The effect of growth hormone on the expression of the *IGF-I* gene has been proved in various animals. López-Fernández et al. injected recombinant human growth hormone into aging rats for 7 days continuously, and the expression of the *IGF-I* gene was restored to the level of adult rats [50]. After Pedroso et al. injected recombinant growth hormone into salmon, the expression level of *IGF-I* gene mRNA in the liver increased significantly [51]. In this experiment, the expression trend of *IGF-I* gene mRNA in the liver of male and female turtles was similar with the continuous injection of growth hormone. In the first week, the expression of *IGF-I* gene mRNA in the liver of turtles was upregulated. The turtles then adapted to the injection amount of exogenous growth hormone. The expression amount of *IGF-I* gene mRNA in the liver required a lot of *SS* to balance its growth rate; the male turtles adapted to the growth hormone, and after two weeks, the turtles adapted to the injection amount of exogenous growth hormone, the *IGF-I* gene in the liver. The continuous upregulation of mRNA may be due to the accumulation of growth hormone in the body after the third week, the sensitivity of the turtles to growth hormone being reduced, and the turtles adapting to the injection amount of exogenous growth hormone. According to the analysis of the changing trend of the mRNA of *SS*, *GHR*, and the *IGF-I* gene in the four weeks after injection, when the expression of *SS* gene mRNA in the brain was downregulated, the expression of *GHR* and *IGF-I* gene mRNA in the liver was upregulated. When the expression of *SS* gene mRNA in the brain was upregulated, the expression of *GHR* and *IGF-I* gene mRNA in the liver was downregulated at the same time, suggesting that the expression level of the *SS* gene in the brain was related to the expression level of *GHR* and *IGF-I*. After the injection of growth hormone, although the increase in *SS* gene expression inhibited the secretion of GH, the exogenous growth hormone was able to take the place of GH synthesized in vivo and *GHR*, which accelerated the growth of the turtles. Although the expression of the *SS* gene increased in the early stage of injection, the expression of the *SS* gene decreased slowly in the late stage of injection, which may indicate that the adaptability of turtles to exogenous growth hormones improved. Our laboratory measured the concentration of *IGF-I* in the blood of the Reeves’ turtle. The concentration of *IGF-I* in the blood of the yellow-feathered broiler is approximately three times that of the Reeves’ turtle, and the content of *IGF-I* in the blood of the blue pond pig is about ten times that of the Reeves’ turtle [3]. In combination with this experiment, it was found that the *IGF-I* gene can be expressed in a large amount in turtles, so it is suggested that the slow growth of the Reeves’ turtle may be related to its low expression level of growth hormone.

## 5. Conclusions

Before this study was performed, there was no data available on *SS* in turtles, and no data regarding the growth-axis-related genes of turtles injected with exogenous growth hormone. In this study, we found that exogenous growth hormones can effectively stimulate the changes in genes related to the growth axis, which may help us to understand the molecular mechanism of the growth of turtles. The results in this study indicated that *SS* gene mRNA expression was the highest in the brain and the lowest in the liver. *GHR* gene mRNA and *IGF-I* gene mRNA are expressed in the brain, hypophysis, liver, kidney, spleen, stomach, intestine, and other tissues. In males, *GHR* gene mRNA expression is the highest in the brain, while in females, *IGF-I* gene mRNA expression is the highest in the liver. In females, *IGF-I* gene mRNA expression is higher in the liver, kidney, and spleen than that in the male. At the early stage of the injection, *SS* gene expression increased, but it later slowly reduced. This may suggest the adaptability of the Reeves’ turtle to exogenous growth hormone. At the same time, exogenous growth hormone can be recognized by the *GHR* gene and can promote the expression of the *IGF-I* gene. The experiments showed that the injection of recombinant human growth hormone affected the expression of *SS*, *GHR*, and *IGF-1* genes, and promoted the growth of the Reeves’ turtle. This provides a theoretical basis for further research on the molecular biology of tortoises and turtles, and guidance on breeding production.

## Data Availability

The data that support the findings of this study are available from the corresponding author upon reasonable request.

## References

[1] Omeyer L.C.M., Fuller W.J., Godley B.J., Snape R.T.E. (2019). The effect of biologging systems on reproduction, growth and survival of adult sea turtles. Mov. Ecol..

[2] Congdon J.D., Nagle R.D., Kinney O.M., van Loben Sels R.C., Quinter T., Tinkle D.W. (2003). Testing hypotheses of aging in long-lived painted turtles (*Chrysemys picta*). Exp. Gerontol..

[3] Congdon J.D., Nagle R.D., Kinney O.M., van Loben Sels R.C. (2001). Hypotheses of aging in a long-lived vertebrate, Blanding’s turtle (*Emydoidea blandingii*). Exp. Gerontol..

[4] Omeyer L.C.M., Fuller W.J., Godley B.J., Snape R.T.E. (2018). Determinate or indeterminate growth? Revisiting the growth strategy of sea turtles. Mar. Ecol. Prog. Ser..

[5] Frýdlová P., Mrzílková J., Šeremeta M., Křemen J. (2021). Determinate growth is predominant and likely ancestral in squamate reptiles. Proc. R. Soc. B Biolpgical Sci..

[6] Frýdlová P., Mrzílková J., Šeremeta M., Křemen J. (2019). Universality of indeterminate growth in lizards rejected: The micro-ct reveals contrasting timing of growth cartilage persistence in iguanas, agamas, and chameleons. Sci. Rep..

[7] Frýdlová P., Nutilová V., Dudák J., Žemlička J., Němec P., Velenský P., Jirásek T. (2017). Patterns of growth in monitor lizards (Varanidae) as revealed by computed tomography of femoral growth plates. Zoomorphology.

[8] Zhu W., He Y., Ruan Z., Zhang X., Liao L., Gao Y., Lin N., Chen X., Liang R., Liu W. (2020). Identification of the cDNA Encoding the Growth Hormone Receptor (GHR) and the Regulation of GHR and IGF-I Gene Expression by Nutritional Status in Reeves’ Turtle (*Chinemys reevesii*). Front. Genet..

[9] Liu W.-S., Ma J.-E., Li W.-X., Zhang J.-G., Wang J., Nie Q.-H., Qiu F.-F., Fang M.-X., Zeng F., Wang X. (2016). The Long Intron 1 of Growth Hormone Gene from Reeves’ Turtle (*Chinemys reevesii*) Correlates with Negatively Regulated GH Expression in Four Cell Lines. Int. J. Mol. Sci..

[10] Very N.M., Kittilson J.D., Klein S.E., Sheridan M.A. (2008). Somatostatin inhibits basal and growth hormone-stimulated hepatic insulin-like growth factor-I production. Mol. Cell. Endocrinol..

[11] Liu W.S., Li Y. (2006). Studies on the biochemical indices of blood in *Chinemys reevesii*. Acta Hydrobiol. Sin..

[12] Liu W.S., Li Y. (2005). Studies on the micro-structure of hypophysis gland and ultrastructure of adenohypophysis in *Chinemys reevesii*. Acta Hydrobiol. Sin..

[13] Krulich L., Dhariwal A.P.S., Mccann S.M. (1968). Stimulatory and inhibitory effects of purified hypothalamic extracts on growth hormone release from rat pituitary in vitro. Endocrinology.

[14] Brazeau P., Vale W., Burgus R., Ling N., Butcher M., Rivier J., Guillemin R. (1973). Hypothalamic Polypeptide That Inhibits the Secretion of Immunoreactive Hypophysis Growth Hormone. Science.

[15] Pradayrol L., Chayvialle J.A., Carlquist M., Mutt V. (1978). Isolation of a porcine intestinal peptide with C-terminal somatostatin. Biochem. Biophys. Res. Commun..

[16] Lin X.-W., Otto C.J., Peter R.E. (1998). Evolution of neuroendocrine peptide systems: Hormone and somatostatin. Comp. Biochem. Physiol. C Pharmacol. Toxicol. Endocrinol..

[17] Barnett P. (2003). Somatostatin and somatostatin receptor physiology. Endocrine.

[18] Brockway D.F., Griffith K.R., Aloimonos C.M., Clarity T.T., Moyer J.B., Smith G.C., Dao N.C., Hossain M.S., Drew P.J., Gordon J.A. (2023). Somatostatin peptide signaling dampens cortical circuits and promotes exploratory behavior. Cell Rep..

[19] Nagaeva E., Schäfer A., Linden A.M., Elsilä L.V., Egorova K., Umemori J., Ryazantseva M., Korpi E.R. (2023). Somatostatin-Expressing Neurons in the Ventral Tegmental Area Innervate Specific Forebrain Regions and Are Involved in Stress Response. eNeuro.

[20] Tang M., Liu C., Li R., Lin H., Peng Y., Lang Y., Su K., Xie Z., Li M., Yang X. (2023). Spatial and temporal expression pattern of somatostatin receptor 2 in mouse. Chin. J. Biotechnol..

[21] Peluso G., Petillo O., Melone M.A., Mazzarella G., Ranieri M., Tajana G.F. (1996). Modulation of cytokine production in activated human monocytes by somatostatin. Neuropeptides.

[22] Elliott D.E., Li J., Blum A.M., Metwali A., Patel Y.C., Weinstock J.V. (1999). SSTR2A is the dominant somatostatin receptor subtype expressed by inflammatory cells, is widely expressed and directly regulates T cell IFN-γ release. Eur. J. Immunol..

[23] Herszényi L., Mihály E., Tulassay Z. (2013). Somatostatin and gastrointestinal tract. Clinical experiences. Orvosi Hetil..

[24] Kappeler L., Zizzari P., Grouselle D., Epelbaum J., Bluet-Pajot M.T. (2004). Plasma and Hypothalamic Peptide-Hormone Levels Regulating Somatotroph Function and Energy Balance in Fed and Fasted States: A Comparative Study in Four Strains of Rats. J. Neuroendocrinol..

[25] Ben-Shlomo A., Melmed S. (2010). Pituitary somatostatin receptor signaling. Trends Endocrinol. Metab..

[26] Froidevaux S., Eberle A.N. (2002). Somatostatin analogs and radiopeptides in cancer therapy. Biopolymers.

[27] Strosberg J., Kvols L. (2010). Antiproliferative effect of somatostatin analogs in gastroenteropancreatic neuroendocrine tumors. World J. Gastroenterol..

[28] Gadelha M.R., Bronstein M.D., Brue T., Coculescu M., Fleseriu M., Guitelman M., Pronin V., Raverot G., Shimon I., Lievre K.K. (2014). Pasireotide versus continued treatment with octreotide or lanreotide in patients with inadequately controlled acromegaly (PAOLA): A randomised, phase 3 trial. Lancet Diabetes Endocrinol..

[29] McLean E., Donaldson E.M. (1993). The Role of Growth Hormone in the Growth of Poikilotherms. Endocrinol. Growth Dev. Metab. Vertebr..

[30] Reindl K., Sheridan M.A. (2012). Peripheral regulation of the growth hormone-insulin-like growth factor system in fish and other vertebrates. Comp. Biochem. Physiol. Part A Mol. Integr. Physiol..

[31] Shafik B.M., Kamel E.R., Mamdouh M., Elrafaay S., Nassan M.A., El-Bahy S.M., El-Tarabany M.S., Manaa E.A. (2022). Performance, Blood Lipid Profile, and the Expression of Growth Hormone Receptor (GHR) and Insulin-Like Growth Factor-1 (IGF-1) Genes in Purebred and Crossbred Quail Lines. Animals.

[32] Pierce A.L., Fox B.K., Davis L.K., Visitacion N. (2007). Prolactin receptor, growth hormone receptor, and putative somatolactin receptor in Mozambique tilapia: Tissue specific expression and differential regulation by salinity and fasting. Gen. Comp. Endocrinol..

[33] Sparkman A.M., Schwartz T.S., Schwartz T.S., Madden J.A., Boyken S.E., Ford N.B., Serb J.M., Bronikowski A.M. (2012). Rates of molecular evolution vary in vertebrates for insulin-like growth factor-1 (IGF-1), a pleiotropic locus that regulates life history traits. Gen. Comp. Endocrinol..

[34] Denley A., Cosgrove L.J., Booker G.W., Wallace J.C., Forbes B.E. (2005). Molecular interactions of the IGF system. Cytokine Growth Factor Rev..

[35] Liu Y., Zhu X., Chen Y. (2005). Age and growth in reeves’ turtle Chinemys reebesii Gray. J. Dalian Fish. Univ..

[36] Yang Y., Ren Z.Y., Su C.B., Wang R.Z., Ma W.B. (2004). Diagnosis and treatment of pituitary growth hormone adenoma. Chin. J. Neurosurg. Dis. Res..

[37] Jiang Q., Lian A., He Q. (2016). Dopamine inhibits somatolactin gene expression in tilapia pituitary cells through the dopamine D2 receptors. Comp. Biochem. Physiol. Part A Mol. Integr. Physiol..

[38] Ye X., Li W.S., Lin H.R. (2007). Progress of research on fish somatostatin and receptor. J. Fish. China.

[39] Li C.J., Wei Q.W., Zhou L., Cao H., Zhang Y., Gui J.F. (2009). Molecular and expression characterization of two somatostatin genes in the Chinese sturgeon, *Acipenser sinensis*. Comp. Biochem. Physiol. Part A Mol. Integr. Physiol..

[40] Kamegai J., Unterman T.G., Frohman L.A., Kineman R.D. (1998). Hypothalamic/hypophysis-axis of the spontaneous dwarf rat: Autofeedback regulation of growth hormone(GH) includes suppression of GH releasing-hormone receptor messenger ribonucleic acid. Endocrinology.

[41] Hu B., Hu S., Yang M., Liao Z., Zhang D., Luo Q., Zhang X., Li H. (2019). Growth Hormone Receptor Gene is Essential for Chicken Mitochondrial Function In Vivo and In Vitro. Int. J. Mol. Sci..

[42] Won E.T., Douros J.D., Hurt D.A., Borski R.J. (2016). Leptin stimulates hepatic growth hormone receptor and insulin-like growth factor gene expression in a teleost fish, the hybrid striped bass. Gen. Comp. Endocrinol..

[43] Mertani H.C., Morel G. (1995). In situ gene expression of growth hormone (GH) receptor and GH binding protein in adult male rat tissues. Mol. Cell. Endocrinol..

[44] Burnside J., Liou S.S., Zhong C., Cogburn L.A. (1992). Abnormal growth hormone receptor gene expression in the sex-linked dwarf chicken. Gen. Comp. Endocrinol..

[45] Ymer S.I., Herington A.C. (1992). Developmental expression of the growth hormone receptor gene in rabbit tissues. Mol. Cell. Endocrinol..

[46] Yakar S., Setser J., Zhao H., Stannard B., Haluzik M., Glatt V., Bouxsein M.L., Kopchick J.J., LeRoith D. (2004). Inhibition of growth hormone action improves insulin sensitivity in liver IGF-1–deficient mice. J. Clin. Investig..

[47] Kim I., Jin E.J., Baik K., Park C.H., Kim W.K., Kang C.W., Ko Y., Jang I., Choi W.S., Lee C.Y. (2008). Expression and secretion of the insulin-like growth factor system components by pig liver cells. Asian Australas. J. Anim. Sci..

[48] de Brun V., Meikle A., Casal A., Carriquiry M., Menezes C., Forcada F., Sosa C., Abecia J.A. (2016). Hepatic Expression of Insulin-Like Growth Factor-1 in Underfed Pregnant Ewes. J. Agric. Ence Technol. A.

[49] Schmid A.C., Lutz I., Kloas W., Reinecke M. (2003). Thyroid hormone stimulates hepatic IGF-I mRNA expression in a bony fish, the tilapia *Oreochromis mossambicus*, in vitro and in vivo. Gen. Comp. Endocrinol..

[50] López-Fernández J., Sánchez-Franco F., Velasco B., Tolón R.M., Pazos F., Cacicedo L. (1996). Growth hormone induces somatostatin and insulin-like growth factor I gene expression in the cerebral hemispheres of aging rats. Endocrinology.

[51] Pedroso F.L., Fukada H., Masumoto T. (2009). In vivo and in vitro effect of recombinant salmon growth hormone treatment on IGF-I and IGFBPs in yellowtail Seriola quinqueradiata. Fish. Sci..

